# Evaluation of Acute and Subchronic Oral Toxicity of Copra Meal Hydrolysate: A Novel Candidate for Prebiotic in Sprague Dawley Rats

**DOI:** 10.1155/jt/7235371

**Published:** 2025-06-05

**Authors:** Jiraporn Tangthong, Francis Ayimbila, Massalin Nakphaichit, Suttipun Keawsompong

**Affiliations:** ^1^Department of Biotechnology, Faculty of Agro-Industry, Kasetsart University, Bangkok 10900, Thailand; ^2^Center for Advanced Studies for Agriculture and Food, KU Institute of Advanced Studies, Kasetsart University (CASAF, NRU-KU), Bangkok 10900, Thailand

**Keywords:** acute toxicity, copra meal hydrolysate, prebiotic, subchronic toxicity

## Abstract

Copra meal hydrolysate (CMH) with high protein and mannooligosaccharides (MOS) was derived by β-mannanase hydrolysis. CMH has been shown to elicit health benefits via prebiotic properties. However, a systematic examination of its safety is required before effective utilization. This study assessed CMH oral acute toxicity at a single dose of 2000 mg/kg for 14 consecutive days, while a subacute toxicity test was conducted by daily oral administration of CMH at doses of 0.25, 0.5 and 1.0 mg/kg for 90 days using Sprague Dawley rats and following OECD guidelines 423 and 408. The acute toxicity study showed that the LD_50_ of CMH was over 2000 mg/kg since no mortality or abnormal clinical signs were observed at this dose. The subacute toxicity results showed that CMH did not induce any abnormalities in body weight, food and water consumption, clinical signs, haematology, clinical chemistry, organ weight and necropsy. Significant changes in some of the parameters were observed but most were not treatment-related and had no effect on animal health. No toxicity-related microscopic findings were recorded in the examined tissues (lung, heart, liver, spleen and kidneys). Oral administration of CMH had a ‘no observed adverse effect level (NOAEL)' of 1.0 mg/kg for both male and female Sprague Dawley rats. CMH demonstrated a high level of safety in animal studies and can be considered a safe prebiotic substance for use in the food and nutraceutical industries.

## 1. Introduction

**Figure 1:**
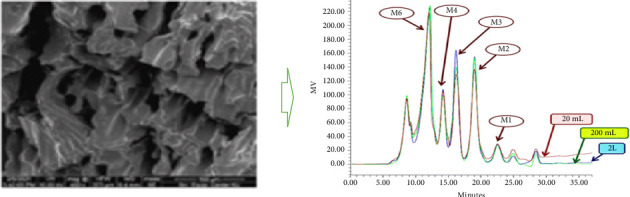
Scanning electron microscopy (SEM) images of hydrolysed copra meal and HPLC analysis of MOS in copra meal hydrolysate; source [1].

Copra meal hydrolysate (CMH) is a prebiotic functional ingredient produced by β-1,4-mannanase hydrolysis of defatted or nondefatted copra meal (CM). CMH has a reduced fibre content consisting of lower levels of neutral detergent fibre (NDF), acid detergent fibre (ADF), cellulose, hemicellulose and lignin [1]. The fibre content, mainly galactomannan, is broken down into mannooligosaccharides (MOS), mostly mannobiose, mannotriose, mannotetraose, mannopentaose and mannohexaose and reducing sugars such as mannose and glucose (Figure 1) [2–5]. The MOS generated in CMH are composed of α- and β-forms of galactose or glucose [6]. Hydrolysed CM has an improved protein value compared to the original CM [1], with the β-MOS structure stimulating innate immune responses in a mouse macrophage model in vitro [7]. MOS obtained from CM and palm kernel cake (PKC) mannans displayed significant iron chelating, radical scavenging and antiglycating activities [7]. Previous research reported that MOS derived from CM had antitumour effects [8].

The International Scientific Association of Probiotics and Prebiotics (ISAPP) defines a dietary prebiotic as a ‘selectively fermented ingredient that results in specific changes in the composition and/or activity of the gastrointestinal microbiota, thus conferring benefit (s) upon host health' [9]. CMH is stable in the upper human gastrointestinal tract and promotes beneficial gut microbes [5, 10]. An in vitro human faecal fermentation showed that CMH promoted the growth of *Lactobacilli* and *Bifidobacteria* similar to fructooligosaccharides (FOS). Induction of two beneficial microbes enhanced short-chain fatty acid (SCFA) production and also suppressed pathogenic bacteria such as Salmonella, *Escherichia coli*, *Staphylococcus aureus* and *Shigella dysenteriae* [10]. The most established prebiotics for human consumption include inulin and its derived FOS, galactooligosaccharides (GOS) and lactulose [11]. Most prebiotics require an oral dose of 3–5 g/d to confer gut health benefits depending on prebiotic structure. Daily dosages are suggested at around 5 g for FOS, GOS and plant sources of prebiotics [12]. Consuming 3 g of CMH per day improves the gut microbiome by increasing beneficial bacteria [5]. Consumption of 3 g of CMH per day by 60 healthy people increased microbial diversity and richness. The ratio of *Firmicutes* to *Bacteroidetes* reduced and the growth of fibre-degrading bacteria, particularly human colonic *Bacteroidetes* increased, while *Bifidobacteriaceae* and immunoglobulin A (IgA) in stool samples improved. The biological activities of CMH have been previously studied, but no toxicological information is available. CMH, created using a fed-batch method, showed improved protein and MOS levels as a novel functional ingredient [13]. CMH also has prebiotic characteristics that benefit the human gut microbiome [5]. Various health benefits of using CM or the MOS produced from CM have been previously documented. This research assessed and forecasted the toxicity of CMH in animals to define safe dosages. The acute and subchronic oral toxicity of CM hydrolysate in Sprague Dawley (SD) rats was investigated based on single-dose acute oral toxicity and repeated dose 90-day oral toxicity studies, as described in OECD guidelines 423 and 408 [1, 14].

## 2. Material and Methods

### 2.1. CM Hydrolysate Preparation

CM residue was collected from the coconut milk-manufacturing factory after coconut milk extraction. The residue was dried at 60°C for 2–4 h before mixing and processing to a particle size of 0.5 mm. A Soxhlet apparatus (Gerhardt Soxtherm Multistat/SX PC, Königswinter, Germany) using petroleum ether was used to extract the residual oil from the CM [10]. CMH was obtained by enzyme hydrolysis of defatted CM by β-mannanase from *Bacillus circulans* NT 6.7 at a concentration of 16.52 U/mL, substrate concentration (15%) and reaction time (12 h) [3]. The CMH was lyophilized and prepared for further study.

### 2.2. Animals

Healthy young male and female SD rats with body weight 200 ± 10% g were obtained from the Office of Laboratory Animal Production, NLAC, Mahidol University, Thailand. The animals were kept under standard conditions of 12:12 (day:night cycles) at 22 ± 2°C and 30%–70% relative humidity, fed with standard feed for rats: 082 (Perfect Companions, Thailand) and given 5–7 ppm chlorinated water ad libitum. One hundred animals (50 males and 50 females) were randomized into five groups as the control (distilled water), three treatments and the satellite group (1.00 mg/kg body weight of test item and withdrawal for 14 days after the feeding period). The three treatments consisted of low dose (0.25 mg/kg body weight of test item), medium dose (0.50 mg/kg body weight of test item) and high dose (1.00 mg/kg body weight of test item). All the animals were acclimated for at least 5 days before the study. Guidelines of ‘Guide for the care and use of laboratory animals' were strictly followed. The research study was approved by the National Laboratory Animal Center Animal Care and Use Committee (NLAC-ACUC), Mahidol University, Thailand [15], under NLAC-ACUC No. RA2016-12 ‘Acute oral toxicity test of copra meal hydrolysate' and NLAC-ACUC No. RA2016-13 ‘3.4 Sub-chronic oral toxicity test of copra meal hydrolysate'.

### 2.3. Acute Oral Toxicity

**Figure 2:**
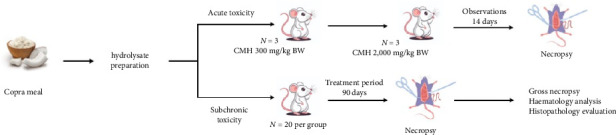
Schematic representation of the experimental design used to assess the acute and subchronic oral toxicity of copra meal hydrolysate.

The acute oral toxicity test complied with the Organisation for Economic Cooperation and Development (OECD) guidelines for the testing of chemicals 423, acute toxic class method (Figure 2). The study comprised male and female SD rats using a stepwise procedure at dosages of 5, 50, 300 and 2000 mg/kg body weight. The starting dose for the CMH was 300 mg/kg body weight. The test sample was administered in a single dose by gavage to three rats. The rats were observed for toxic effects for the first 30 min with special attention given during the first 4 h (unusual respiratory pattern, excretions and autonomic activity) periodically during the first 24 h. If no signs of toxic effects or mortalities were observed within the first 24 h, the next dosage level (2000 mg/kg body weight) was administered to another three rats. If all the rats survived, they were clinically observed once daily for 14 days. After the observation period (14 days), all the animals were subjected to a full, detailed gross necropsy which included careful examination of the external surface of the body, all orifices and the cranial, thoracic and abdominal cavities, brain and skin layer.

### 2.4. Subchronic Oral Toxicity

The repeated dose study was compliant with OECD guideline 408: repeated dose 90 days oral toxicity study in rodents (Figure 2). Dosage levels of 0.25 (low dose), 0.50 (medium dose) and 1.0 mg/kg body weight (high dose) were used for the study. All the rats were randomized into five groups (10 males, 10 females per group). The three treatment groups were treated with 0.25, 0.50 and 1.0, and the satellite group was treated with 1.0 mg/kg body weight of CMH. The control group was treated with distilled water. All the rats were administered by gavage daily for 90 days. The administration volumes were calculated based on recent weekly body weights and adjusted weekly to maintain the targeted dosage levels. Each rat was administered at a constant volume of 2 mL/100 g body weight. Clinical observations including changes in skin and fur, eyes and mucus membrane, respiratory, circulatory and behaviour patterns, tremors, convulsion, salivation, diarrhoea, sleep and coma were investigated for all the experimental rats after the administration of doses and thereafter once a day for 90 days. Body weights and feed and water consumption were recorded weekly. The rats in the satellite group were followed up for observation without treatment for 14 days. On the last day of administration, the rats were subjected to overnight fasting (water ad libitum) and then euthanized using CO_2_ inhalation.

### 2.5. Clinical Observation

All the animals were observed daily for clinical signs, and the clinical conditions were recorded. The observed signs included but were not limited to changes in skin, fur, eyes and mucous membranes, and occurrence of secretions and excretions and autonomic activity (e.g., lacrimation, pupil size, piloerection and unusual respiratory patterns). Changes in gait, posture, responses to handling and the presence of colonic or tonic movements, stereotypy or bizarre behaviour (e.g., self-mutilation, walking backwards) were also recorded.

### 2.6. Gross Necropsy

After the experiment, the animals were euthanized using the CO_2_ inhalation method. Blood samples were collected by cardiac puncture for exsanguination, and the carcass was examined for gross necropsy by studying the external surface of the body, the cranial, thoracic and abdominal cavities and their contents.

### 2.7. Organ Weight

The absolute and relative organ weights including brain, heart, kidneys, liver, ovaries, spleen, thymus and uterus (including cervix and oviducts) were measured for adrenals.

The relative organ weights were calculated according to the following formula:

(1) $$\begin{array}{c} \text{Relative organ weight }\left(\%\right)=\left[\text{organ weight }\left(g\right)/\text{body weight }\left(g\right)\right]\times 100. \end{array}$$

### 2.8. Clinical Biochemical Analysis

A clinical biochemical analysis was performed on the serum obtained after the centrifugation of total blood (without anticoagulant) using a CobasC311 automated blood analyser (Roche, Switzerland) for total protein, cholesterol, triglyceride, alanine transaminase (ALT), aspartate transaminase (AST), alkaline phosphatase, total bilirubin, albumin, globulin, creatinine, blood urea nitrogen, uric acid and glucose.

### 2.9. Haematological Analysis

Blood samples were collected via cardiac puncture into test tubes for haematological and clinical biochemical analysis. The haematological analysis was performed using an automated analyser (IDEXX ProCyte Dx™, USA) to determine red blood cells (RBCs), haemoglobin (HBG), haematocrit (HCT), mean corpuscular volume (MCV), mean corpuscular haemoglobin (MCH), mean corpuscular haemoglobin concentration (MCHC), platelets (PLT), white blood cells (WBCs), neutrophils (Neut), lymphocytes (Lymph), monocytes (Mono), eosinophils (Eosi) and basophils (Baso).

### 2.10. Histopathological Evaluation

A full histopathological evaluation was carried out on the preserved organs and tissues of all the animals in the control and high-dose groups. Haematoxylin and eosin (H&E)–stained sections of the animals were prepared for the histopathological assessment. The tissue sections were examined under a light microscope to detect pathological alterations in the vital organs (spleen, kidneys, liver, heart and lung).

### 2.11. Statistical Analysis

Quantitative results were expressed as mean ± standard deviation. Data were statistically analysed using SPSS Statistical software version 18.0.0, with one-way analysis of variance (ANOVA) employed to compare the mean values of the control and treatment groups. A significant difference was considered at the 0.05 level.

## 3. Results

### 3.1. CMH Characteristics

An oligosaccharide concentration of 14.34 ± 0.11 mg/mL was obtained under experimental conditions of enzyme concentration (16.52 U/mL), substrate concentration (15%) and reaction time (12 h). The obtained CMH consisted of mannobiose (M2), mannotriose (M3), mannotetraose (M4), mannopentaose (M5) and mannohexaose (M6) with concentrations (mg/mL) of M3(6.28) > M2(6.08) > M4 (3.63) > M5 (2.75) > M6 (less than 0.03). Our results concurred with a previous report [3].

### 3.2. Acute Oral Toxicity

Oral administration of CMH at 2000 mg/kg body weight resulted in no deaths of rats over 24 h and 14 days, with no evidence of toxicity, behavioural or physiological abnormalities. The body weight gain during the observation period among the treated animals was comparable to their respective controls, with no sex-related variations seen. The necropsy exams indicated no harm to organs or tissues. The lethal dose, 50% (LD_50_), of CMH was greater than 2000 mg/kg body weight and CMH did not show acute toxicity.

### 3.3. Subchronic Toxicity Study

#### 3.3.1. Survival and Clinical Observations

Throughout the 13-week dosing period, all animals in the control and treated dose groups survived. Clinical and ophthalmological tests revealed no abnormal signs or symptoms such as changes in skin and hair, eyes and mucus membranes, respiratory, circulatory and behavioural patterns, tremors, convulsion, salivation, diarrhoea, sleep and coma in male and female rats in any group at 0.25, 0.5 and 1.0 mg/kg doses. Necropsies also revealed no abnormalities that could be attributed to the treatment, with no significant treatment-related changes in rat behaviour or locomotor activity during the experiment.

#### 3.3.2. Body Weight Gain, Feed and Water Consumption

**Figure 3:**
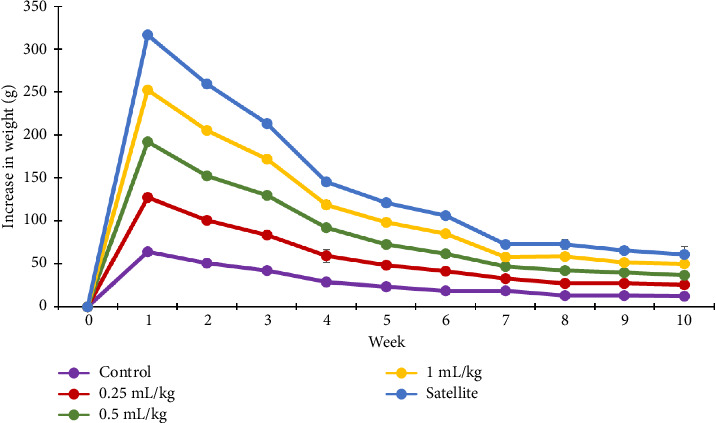
Mean weight increase of the control and treated male rats in the subchronic oral toxicity study.

**Figure 4:**
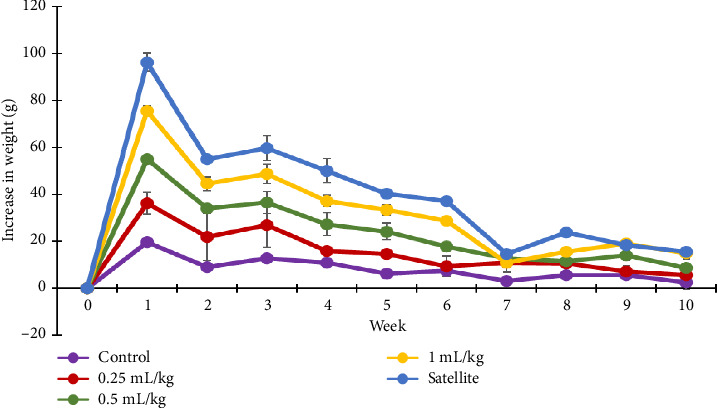
Mean weight increase of the control and treated female rats in the subchronic oral toxicity study.

No symptoms of toxicity or mortality were recorded in either sex after receiving CMH extracts (1.0, 0.5 and 0.25 mg/kg) for 90 days. Water and food consumption of the treated animals did not significantly differ compared to the control. Over the same time period, no significant differences in percentage body weight gain were detected between the control and treatment groups (Figures 3 and 4). How CMH ingestion impacts the body weight of animals is important for evaluating toxicity. A change in overall body weight or the organ–body weight ratio indicates a problem with the normal functioning of the organs [16]. Male and female rats in both the control and treated groups showed gradual increases in body weight over the 13-week treatment period, as indicated in Table 1. The highest body weight rises in the rats occurred after the first week and continued to reduce until the last week, similarly among the treatments (Figures 3 and 4), indicating that CMH had no effect on weight changes.

#### 3.3.3. Organ Weight

The mean organ weights of male and female rats in the high-dose group (1.0 mg/kg body weight) were not significantly different from the control group. Dosages of 0.25 mg, 0.50 mg and 1.0 mg/kg had no effect on the size of essential organs such as the liver, lung, heart, brain, adrenal and testis in female rats (Table 2). The extract was nontoxic to the examined organs, with no reduction in the relative body or essential organ weight in the treated animals at any tested doses.

#### 3.3.4. Clinical Pathology—Haematological Investigations

The haematological examinations performed at the end of the dosing period on week 13 and at the end of the recovery period revealed significant changes (*p* < 0.05) in the values of the various parameters studied compared to the respective controls (Table 3). However, the observed increase/decrease in values was within normal biological and laboratory limits, indicating that the effect was not dose-dependent. In males, the 0.25 mg/kg CMH-treated rats had significantly lower MCHC, while the 0.50 mg/kg CMH-treated rats had significantly lower WBC and MCV concentrations than the respective control groups. Plateletcrit (PCT), platelet distribution width (PDW) and MCHC in the 0.50 mg/kg CMH-treated groups were significantly higher than those in the control group. At 1.0 mg/kg CMH, PCT was significantly higher and neutrophils (N) were significantly lower than in the control group. Mean platelet volume (MPV), red blood cell distribution width (RDW) and PDW were significantly higher in the satellite group compared to the control group, while basophil (B) was significantly lower. WBC, MCV, MCHC, RDW, MPV, PDW and B had no treatment-related effects, unlike PCT and N. In female rats, MCV was significantly lower in the 0.25 mg/kg and 0.5 mg/kg treated groups than in the control groups. PDW was significantly higher in 1.0 mg/kg treated rats, while MCV was significantly lower. When the 0.5 mg/kg treated animals were compared to the control animals, PDW, MPV and PCT were significantly higher. PDW was significantly higher in the satellite group and B was significantly lower in the control group. PDW, MPV, PCT and WBC were inferred to be unrelated to treatment, while MCV was a treatment-related side effect.

### 3.4. Blood Chemistry

The results of the clinical biochemical analysis are shown in Table 4. Male rats showed a significant decrease (*p* < 0.05) in total protein, albumin, globulin, alkaline phosphatase, AST, creatinine, glucose, total cholesterol and triglyceride levels in CMH (1.0 mg/kg) treated animals compared to the control animals. In female rats, alkaline phosphatase, blood urea and glucose levels were significantly higher (*p* < 0.05) than those in the control group. Male rats administered at 1.0 mg/kg showed changes in the biochemical indicators AST and ALT in their blood. At 1.0 mg/kg dosage, alteration in AST was detected in female rats. AST and ALT are two key enzymes that indicate injury of hepatocytes and the liver [17]. However, these significant changes in blood chemistry results were not regarded as a treatment-related toxicity effect.

### 3.5. Necropsy and Histopathological Evaluation

The necropsy and histopathological results of CMH-treated animals are summarized in Table 5. The histopathological findings of adrenals, brain, heart, pancreas, spleen, testes and ovaries from CMH (1.0 mg/kg)-treated animals did not show any necropsy or histological aberrations (Table 6). However, a proteinaceous plug at the urinary bladder, grey consolidation and enlargement at the lung, mild hydronephrosis with moderate calcification at the left kidney, gas distension in the colon, mild contusion of the penis and blood clot urogenital orifice, watery green diarrhoea, mild petechial haemorrhage at the right thymus and hydrometra at the uterus were observed in the CMH-treated and control animals. All the lesions in the liver, lung and heart in the control and high dosage animals were considered spontaneous/incidental findings due to low frequency or similar incidence and/or severity in the control group.

## 4. Discussion

CM, a by-product of the coconut milk industry, is primarily used in animal feed due to its low cost and nutritional composition. However, the main content of CM, galactomannan, reduces its potential as a protein and energy source [18]. Beta-mannanase from *Bacillus circulans* NT 6.7 increases the quality of animal feed by breaking down galactomannan to release oligosaccharides [1]. CMH induces prebiotic effects by promoting intestinal flora diversity and enhancing lactic acid production. A daily dose of CMH led to potentially beneficial effects on gut health for healthy individuals [5]. The assessment and evaluation of the toxic properties of natural compounds are the first steps in screening a new functional ingredient to improve health. CMH has shown pharmacologically advantageous properties, but comprehensive information about the toxic effect of this prebiotic is lacking. This study assessed the acute and subchronic toxicity of CHM in SD rats.

The exposure of rats to a single dose of CMH (up to 2000 mg/kg) did not reveal any mortality or adverse clinical signs during 14 days of acute oral toxicity testing. Body weight gain during the observation period among the treated animals was comparable to their respective controls, with no sex-related variations. The LD_50_ of orally administered CMH exceeded 2000 mg/kg in both sexes. Acute toxicity testing on CMH has not been recorded in the literature. If a substance shows no clinical indications of toxicity or causes mortality after 14 days of acute oral toxicity testing, it is termed nontoxic [19]. Therefore, the ‘no observed adverse effect level (NOAEL)' was considered to be 2000 mg/kg body weight per day for SD rats. The results provide insights into the nontoxicity of CMH and may help in the formulation of an appropriate dosage for nutrition or clinical uses.

Subchronic toxicity studies are critical in assessing the safety profile of drugs or substances because data gained from acute toxicity studies are insufficient to assess clinical implications [20, 21]. Most plant-derived prebiotics show no acute toxicity [22, 23] but repeated dose toxicity is required to determine the potential toxic effects of a substance after repeated administration over a limited period. This study investigated the effects of CMH after oral administration in rats for 13 weeks. The results of the repeated dosage toxicity tests were used to determine the toxic effects and target organ identification and impacts on the physiology as a haematological metabolic profile and histological history of the animal. Regulatory organizations mandate these tests to assess the toxicological potential of substances [1, 14]. Chronic administration of 0.25 mg, 0.50 mg and 1.00 mg/kg CMH for 13 weeks showed no significant changes in animal behaviour, body weight, food and water consumption and necropsy findings. Male rats in the high-dose group showed significant increases in the weights of their left and right kidneys, testis and epididymis, while the mean organ weights of female rats did not differ significantly from the control group. The sizes of vital organs such as liver, lung, heart, brain, adrenal and testis were unaffected by CMH. A high dose of CMH did not cause any significant changes in the organs of treated rats. The increased organ weights (left and right kidney, testis and epididymis) in male rats were statistically significant but assumed to be toxicologically irrelevant because they were of minor magnitude or within the current reference ranges for SD rats of the same sex and age [20, 24]. They were not linked to any other clinical indications, and there was no evidence of a pathological problem.

The haematological analysis and clinical biochemical parameter results were used to assess the risk of haematological system alterations. Preclinical data on substance toxicity have a higher predictive value for clinical toxicity [25]. Repeated administration of CMH over 13 weeks caused minor but statistically significant haematological changes in blood parameters in both male and female rats, as increases in HBG, RBC count and HCT levels. Low MCHC values are associated with iron deficiency [26] and may be the cause of low HBG concentration. MCV is also linked to individual RBCs, whereas HBG and RBC are related to the total population of RBCs in the blood [27]. CMH had no effect on the incorporation of HBG into RBCs or the morphology and osmotic fragility of the RBCs produced, indicating that it had no toxic effects on the oxygen-carrying capacity of the blood system. The WBCs were lower in male rats and higher in female rats treated with CMH but above the abnormal level and this was assumed to be toxicologically irrelevant, indicating that CMH did not trigger immune responses associated with the destruction or impaired production of WBCs.

After 13 weeks, serum biochemical parameters such as blood urea nitrogen and creatinine in CMH-treated rats were significantly altered. Creatinine is formed in muscles by the hydrolysis of creatine and phosphocreatine. When renal function is compromised, creatine levels rise [28]. BUN, a by-product of protein metabolism, is primarily produced in the liver. A high BUN level indicates severe kidney function impairment [29]. These values were significantly altered, but they remained within the normal range [20, 30]. Urinalysis results including urine volume, haematological and serum biochemical tests and necropsy revealed no abnormal signs of kidney function. Apart from the increase in kidney weight in CNH-treated rats, they showed no other changes, supporting these biochemical findings.

The evaluation of protein levels in the blood along with glucose metabolism, protein synthesis, biliary secretion or liver function can reveal whether the liver cells are damaged. The enzymes ALT and AST are markers of liver function [31, 32]. ALT, a cytoplasmic enzyme found in abundance in the liver, is significantly elevated during hepatocellular insult [33, 34]. Many tissues including the heart, skeletal muscles, liver, kidneys, pancreas and erythrocytes contain the enzyme AST [35]. AST is released by cells in response to cellular damage or a change in the permeability of their membranes. As a result, ALT is a more sensitive hepatic damage biomarker than AST. The AST and ALT values in the CMH-treated group differed significantly from the control group, but the fluctuations were not gender or dose-dependent and were within normal laboratory ranges [36]. The results also demonstrated that male rats administered 1.0 mg/kg had changes in AST and ALT in their blood, while at 1.0 mg/kg dosage, alterations in AST were also detected in female rats. Numerous macroscopic and microscopic lesions were identified but almost all were spontaneous, oestrous cycle, carbon dioxide effect and incidental findings that were not related to toxicity criteria. These lesions were observed in CMH-treated and control groups and were not thought to be toxicologically significant in relation to CMH exposure.

A recent study revealed the toxicity of a newly synthesized prebiotic molecule, butyl-fructooligosaccharide (B-FOS), formed by the ester bonding of FOS and butyrate [37]. Acute and repeated oral administration of B-FOS at 2000 mg/kg in accordance with OECD protocol test numbers 420 and 407 (28 days repeated administration) resulted in no significant changes in body weight, organ weight, serum biochemical parameters or tissue histology after animal sacrifice. Short-chain FOS (Fossence), produced through a patented process of sucrose biotransformation by the action of enzymes from live microbial cells, is used as a prebiotic in infant formula and conventional foods. In acute and subchronic toxicity studies, Wistar rats were given Fossence at doses of 0, 2000, 5000 and 9000 mg/kg/day for 90 days with no deaths or clinical signs. However, a statistically significant increase in cecum weight (without correlative microscopic change) was observed in all test item-treated groups of males and females and this was thought to be a trophic effect rather than a toxic effect in rats [38–44]. A recent study investigated the toxicity of microencapsulated pomegranate juice (MPJ) in Wistar rats and CD-1 mice. When high doses of 5000 mg/kg were orally administered to rats for 14 days, no deaths or adverse effects occurred, indicating the absence of subacute toxicity. Similarly, 3000 mg/kg MPJ given to CD-1 mice for 90 days showed no subchronic toxicity. MPJ reduced weight gain in both rats and mice. An Alamar blue assay revealed that MPJ had no cytotoxicity in epithelial cell culture, while a histopathological examination of the kidney and liver confirmed the absence of toxicity in CD-1 mice [45].

No deaths, relevant clinical signs or abnormal findings were reported in the acute and subchronic oral toxicity study of CMH, conducted in accordance with OECD 423 and 408 guidelines. A better understanding of the acute and subchronic oral toxicity profile of CMH will be useful for future applications. A 13-week oral dose of CMH (1.0 mg/kg) did not cause toxicological alterations in biochemical, haematological, anatomical or histological investigations. Thus, CMH can be classified as NOAEL for use in animals. However, toxicity in humans cannot always be completely extrapolated from animal studies and further clinical testing is required to establish the safe dosage level in humans.

## 5. Conclusions

An acute and subchronic toxicity study of CMH was performed by oral administration using SD rats as the animal model. Body/organ weights, chemical/haematological analysis and histopathological evaluations showed that CMH administered at 2000 mg/kg for 14 days caused no mortality or evidence of severe toxicity. Thus, the acute oral lethal dose (LD_50_) of CMH was greater than 2000 mg/kg body weight. The subchronic oral toxicity study showed that oral administration of CMH had an NOAEL of 1.0 mg/kg for both male and female rats. CMH revealed no toxicity to the liver in rats at high concentrations (1.0 mg/kg) after 14 days of oral subacute toxicity, as evaluated by haematological, serum biochemical and/or histological studies. CMH demonstrated a favourable safety profile in animal studies and may become a key functional ingredient in promoting human health. However, a novel functional element must be explored and evaluated in detail in terms of toxicity and safety. Therefore, assessing the toxicological consequences of CMH with prospective future applications remains an important element of its risk assessment.

## Figures and Tables

**Table 1:** Body weight changes of rats in the subchronic oral toxicity study of copra meal hydrolysate.

	Group	Body weight (g)		Clinical observation	
		Initial weight	Terminal weight	Behavioural change	Mortality
Male	Control	207.10 ± 6.21	504.77 ± 26.93	No	No
	0.25 mg/kg BW	207.50 ± 5.85	502.89 ± 34.72	No	No
	0.50 mg/kg BW	207.30 ± 4.00	512.34 ± 23.49	No	No
	1.0 mg/kg BW	207.40 ± 5.06	493.06 ± 28.95	No	No
	Satellite	207.30 ± 4.52	527.52 ± 26.69	No	No

Female	Control	196.40 ± 8.47	278.03 ± 5.61	No	No
	0.25 mg/kg BW	197.50 ± 7.65	275.51 ± 12.94	No	No
	0.50 mg/kg BW	197.50 ± 5.70	284.46 ± 10.42	No	No
	1.0 mg/kg BW	197.50 ± 0.8	281.02 ± 8.50	No	No
	Satellite	197.50 ± 5.70	288.27 ± 12.99	No	No

*Note:* Values are mean + SD.

**Table 2:** Organ weights of rats in the subchronic oral toxicity study of copra meal hydrolysate.

**Male**							
**Group**	**Relative organ weights (%)**						
	**Liver**	**Kidney (R)**	**Kidney (L)**	**Heart**	**Lung**	**Spleen**	**Brain**

Control	2.7117 ± 0.12	0.3250 ± 0.02	0.3254 ± 0.02	0.3248 ± 0.02	0.3332 ± 0.04	0.1852 ± 0.01	0.4448 ± 0.02
0.25	2.6842 ± 0.12	0.3311 ± 0.01	0.3237 ± 0.01	0.3287 ± 0.02	0.3281 ± 0.03	0.1792 ± 0.01	0.4503 ± 0.03
0.50	3.0137 ± 0.14	0.3378 ± 0.01	0.3372 ± 0.01	0.3279 ± 0.01	0.3438 ± 0.02	0.1850 ± 0.01	0.4421 ± 0.02
1.0	2.8071 ± 0.27	0.3504 ± 0.02	0.3490 ± 0.02	0.3431 ± 0.02	0.3334 ± 0.04	0.1778 ± 0.01	0.4429 ± 0.03
Satellite	2.8717 ± 0.13	0.3392 ± 0.02	0.3369 ± 0.02	0.3172 ± 0.02	0.3330 ± 0.02	0.1806 ± 0.02	0.4341 ± 0.02

**Group**	**Relative organ weights (%)**						
	**Adrenal (R)**	**Adrenal (L)**	**Testis (R)**	**Testis (L)**	**Epidermis (R)**	**Epidermis (L)**	**Thymus**

Control	0.0054 ± 0.00	0.0058 ± 0.00	0.3832 ± 0.03	0.3982 ± 0.03	0.1449 ± 0.01	0.1434 ± 0.01	0.0666 ± 0.01
0.25	0.0057 ± 0.00	0.0063 ± 0.00	0.4111 ± 0.02	0.4010 ± 0.02	0.1464 ± 0.01	0.1470 ± 0.01	0.0707 ± 0.01
0.50	0.0055 ± 0.00	0.0060 ± 0.00	0.4110 ± 0.04	0.4004 ± 0.02	0.1427 ± 0.01	0.1376 ± 0.01	0.0651 ± 0.01
1.0	0.0058 ± 0.00	0.0064 ± 0.00	0.4299 ± 0.03	0.4306 ± 0.03	0.1509 ± 0.01	0.1567 ± 0.01	0.0652 ± 0.01
Satellite	0.0059 ± 0.00	0.0054 ± 0.00	0.4027 ± 0.03	0.3968 ± 0.02	0.1317 ± 0.02	0.1372 ± 0.01	0.0539 ± 0.01

**Female**							
**Group**	**Relative organ weights (%)**						
	**Liver**	**Kidney (R)**	**Kidney (L)**	**Heart**	**Lung**	**Spleen**	**Brain**

Control	2.6445 ± 0.12	0.3303 ± 0.03	0.3140 ± 0.01	0.3810 ± 0.02	0.4707 ± 0.02	0.2244 ± 0.01	0.7425 ± 0.02
0.25	2.5252 ± 0.21	0.3254 ± 0.01	0.3120 ± 0.02	0.3746 ± 0.02	0.4830 ± 0.04	0.2347 ± 0.02	0.7523 ± 0.03
0.50	2.6302 ± 0.19	0.3331 ± 0.02	0.3242 ± 0.02	0.3672 ± 0.01	0.4797 ± 0.04	0.2205 ± 0.02	0.7218 ± 0.03
1.0	2.6418 ± 0.21	0.3318 ± 0.01	0.3285 ± 0.02	0.3796 ± 0.02	0.5247 ± 0.07	0.2345 ± 0.02	0.7333 ± 0.02
Satellite	2.4931 ± 0.17	0.3162 ± 0.01	0.3090 ± 0.01	0.3611 ± 0.02	0.4785 ± 0.06	0.2129 ± 0.02	0.6524 ± 0.22

**Group**	**Relative organ weights (%)**						
	**Uterus**	**Adrenal (R)**	**Adrenal (L)**	**Ovary (R)**	**Ovary (L)**	**Thymus**	

Control	0.2172 ± 0.05	0.0189 ± 0.02	0.0124 ± 0.00	0.0138 ± 0.00	0.0134 ± 0.00	0.0770 ± 0.01	
0.25	0.1933 ± 0.05	0.0104 ± 0.00	0.0122 ± 0.00	0.0129 ± 0.00	0.0126 ± 0.00	0.0758 ± 0.01	
0.50	0.1958 ± 0.05	0.0107 ± 0.00	0.0119 ± 0.00	0.0135 ± 0.00	0.0146 ± 0.00	0.0725 ± 0.01	
1.0	0.1820 ± 0.03	0.0123 ± 0.00	0.0133 ± 0.00	0.0157 ± 0.00	0.0139 ± 0.00	0.0761 ± 0.01	
Satellite	0.2100 ± 0.04	0.0110 ± 0.00	0.0121 ± 0.00	0.0139 ± 0.00	0.0126 ± 0.00	0.0592 ± 0.02	

*Note:* Values are mean ± SD.

**Table 3:** Haematological analysis of rats in the subchronic oral toxicity study of copra meal hydrolysate.

**Male**						
**Group**	**Parameter**					
	**RBC**	**HGB**	**HCT**	**MCV**	**MCH**	**MCHC**

Control	9.48 ± 0.42	16.2 ± 0.77	50.9 ± 2.74	53.7 ± 0.66	17.1 ± 0.21	21.8 ± 0.45
0.25	9.82 ± 0.31	16.5 ± 0.38	52.8 ± 1.51	53.8 ± 0.65	16.8 ± 0.21	31.2 ± 0.25
0.50	9.29 ± 0.26	16.1 ± 0.57	49.1 ± 1.60	52.9 ± 0.74	17.3 ± 0.39	32.7 ± 0.53
1.0	9.71 ± 0.58	16.3 ± 0.94	51.5 ± 3.28	53.1 ± 0.55	16.8 ± 0.23	31.6 ± 0.28
Satellite	9.76 ± 0.57	16.6 ± 0.70	52.0 ± 3.01	53.3 ± 0.75	17.0 ± 0.39	32.0 ± 0.63

**Group**	**Parameter**					
	**PLT**	**WBC**	**RDW**	**PDW**	**MPV**	**PCT**

Control	871 ± 55.90	6.69 ± 1.69	22.1 ± 0.39	8.0 ± 0.33	6.9 ± 0.27	0.60 ± 0.05
0.25	915 ± 66.75	8.19 ± 1.77	22.4 ± 0.56	8.1 ± 0.23	6.9 ± 0.13	0.63 ± 0.05
0.50	940 ± 69.51	4.58 ± 1.65	22.3 ± 0.63	8.3 ± 0.40	7.1 ± 0.24	0.67 ± 0.06
1.0	944 ± 82.87	7.72 ± 1.60	22.5 ± 0.62	8.2 ± 0.25	7.0 ± 0.17	0.66 ± 0.06
Satellite	882 ± 45.51	6.28 ± 1.13	22.8 ± 0.81	8.4 ± 0.39	7.2 ± 0.23	0.63 ± 0.04

**Female**						
**Group**	**Parameter**					
	**RBC**	**HGB**	**HCT**	**MCV**	**MCH**	**MCHC**

Control	8.94 ± 0.16	16.0 ± 0.30	50.7 ± 0.96	56.7 ± 0.81	17.9 ± 0.29	31.5 ± 0.35
0.25	9.24 ± 0.35	16.3 ± 0.64	52.0 ± 2.19	56.3 ± 0.85	17.7 ± 0.21	31.4 ± 0.20
0.50	9.05 ± 0.40	16.1 ± 0.70	50.4 ± 2.36	55.7 ± 0.88	17.7 ± 0.28	31.9 ± 0.39
1.0	9.04 ± 0.44	16.0 ± 0.75	50.3 ± 2.73	55.7 ± 0.67	17.7 ± 0.17	31.7 ± 0.29
Satellite	8.88 ± 0.32	15.9 ± 0.61	49.8 ± 2.08	56.1 ± 0.84	17.9 ± 0.31	31.9 ± 0.38

**Group**	**Parameter**					
	**PLT**	**WBC**	**RDW**	**PDW**	**MPV**	**PCT**

Control	852 ± 91.32	4.71 ± 0.71	19.5 ± 0.57	7.5 ± 0.14	6.7 ± 0.15	0.57 ± 0.05
0.25	827 ± 44.12	6.71 ± 1.49	19.9 ± 0.83	7.7 ± 0.23	6.8 ± 0.17	0.56 ± 0.02
0.50	946 ± 152.32	5.21 ± 1.38	20.1 ± 0.48	7.9 ± 0.37	7.0 ± 0.24	0.66 ± 0.11
1.0	842 ± 56.93	6.17 ± 1.95	19.6 ± 0.81	8.0 ± 0.30	7.0 ± 0.21	0.59 ± 0.03
Satellite	831 ± 49.21	4.64 ± 0.83	19.9 ± 0.67	8.2 ± 0.37	7.2 ± 0.19	0.59 ± 0.04

**Table 4:** Clinical biochemical analysis of rats in the subchronic oral toxicity study of copra meal hydrolysate.

**Male**						
**Group**	**Parameter**					
	**Total protein**	**Total cholesterol**	**Triglyceride**	**ALT**	**AST**	**Alkaline phosphatase**

Control	7.78 ± 0.97	117.0 ± 14.58	105.5 ± 20.05	52.2 ± 8.98	79.4 ± 8.15	70.9 ± 9.19
0.25	7.70 ± 0.34	107.1 ± 13.06	85.4 ± 17,85	50.1 ± 15.66	75.7 ± 5.42	63.0 ± 5.40
0.50	6.97 ± 0.23	105.7 ± 6.64	88.8 ± 11.06	45.8 ± 5.13	80.0 ± 13.30	64.0 ± 6.02
1.0	6.88 ± 0.27	111.4 ± 7.68	87.7 ± 14.06	62.3 ± 46.01	91.7 ± 55.86	63.0 ± 6.94
Satellite	6.98 ± 0.27	100.0 ± 8.79	69.7 ± 19.83^∗^	43.7 ± 4.96	67.8 ± 6.74	60.0 ± 8.78

**Group**	**Parameter**					
	**Albumin**	**Globulin**	**Creatinine**	**BUN**	**Uric acid**	**Glucose**

Control	4.86 ± 0.38	2.69 ± 0.33	0.34 ± 0.06	19.2 ± 3.25	4.7 ± 1.13	206.1 ± 69.80
0.25	4.93 ± 0.18	2.77 ± 0.26	0.30 ± 0.02	16.8 ± 1.85	4.6 ± 0.71	288.4 ± 34.88
0.50	4.52 ± 0.09	2.44 ± 0.25	0.30 ± 0.03	19.7 ± 2.16	4.0 ± 1.19	192.0 ± 74.70
1.0	4.46 ± 0.13	2.42 ± 0.21	0.26 ± 0.01	18.9 ± 1.63	4.1 ± 0.77	284.7 ± 74.62
Satellite	4.46 ± 0.14	2.48 ± 0.18	0.29 ± 0.02	20.5 ± 3.00	5.3 ± 1.03	307.1 ± 55.87

**Female**						
**Group**	**Parameter**					
	**Total protein**	**Total cholesterol**	**Triglyceride**	**ALT**	**AST**	**Alkaline phosphatase**

Control	7.36 ± 0.20	131.3 ± 22.75	58.1 ± 6.86	47.6 ± 8.25	85.8 ± 12.10	48.6 ± 6.07
0.25	7.52 ± 0.39	143.4 ± 25.71	64.8 ± 13.24	41.6 ± 7.85	85.5 ± 8.91	41.6 ± 7.85
0.50	7.16 ± 0.19	125.8 ± 15.39	57.7 ± 17.11	45.4 ± 6.88	83.2 ± 13.88	45.4 ± 6.88
1.0	7.16 ± 0.28	137.7 ± 20.34	75.8 ± 22.99	46.2 ± 6.97	105.1 ± 39.12	46.2 ± 6.97
Satellite	7.22 ± 0.17	123.4 ± 13.46	65.6 ± 16.70	43.1 ± 9.02	78.4 ± 9.91	43.1 ± 9.02

**Group**	**Parameter**					
	**Albumin**	**Globulin**	**Creatinine**	**BUN**	**Uric acid**	**Glucose**

Control	5.10 ± 0.18	2.20 ± 0.12	0.34 ± 0.03	17.4 ± 2.29	3.3 ± 0.31	110.9 ± 21.07
0.25	5.22 ± 0.23	2.30 ± 0.19	0.33 ± 0.03	17.4 ± 1.90	3.3 ± 1.16	123.5 ± 21.26
0.50	5.07 ± 0.21	2.09 ± 0.10	0.33 ± 0.02	23.0 ± 3.09	3.5 ± 0.50	207.8 ± 80.12
1.0	4.96 ± 0.22	2.20 ± 0.17	0.33 ± 0.03	21.8 ± 1.52	3.1 ± 0.63	200.7 ± 55.71
Satellite	5.22 ± 0.14	2.00 ± 0.09	0.34 ± 0.04	19.3 ± 2.60	4.0 ± 0.87	238.6 ± 76.02

**Table 5:** Effect of copra meal hydrolysate (CMH) on the incidence of necropsy and histopathology.

Sex	Male					Female				
CMH (mg/kg/day)	0	0.25	0.5	1	S	0	0.25	0.5	1	S

Number of animals	10	10	10	10	10	10	10	10	10	10

*External surfaces*										
Body orifices					3					
Integument						1	1			

*Respiratory system*										
Lung	1	1								
Thymus		1	1	1						
Thoracic cavity						1				

*Gastrointestinal system*										
Cecum					1					
Ilium								1		

*Urinary system and endocrine gland*										
Kidneys	2	2		1						1
Urinary bladder										

*Genital system*										
Uterus–cervix/epididymis	4	2	3	1				3		1

**Table 6:** Effect of copra meal hydrolysate (CMH) on necropsy and histopathological findings.

	Control group	CMH (1.0 mg/kg)
Liver	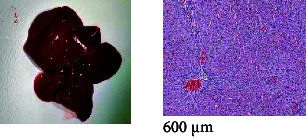	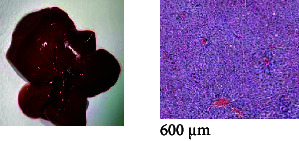

Heart	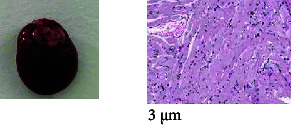	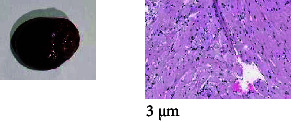

Lung	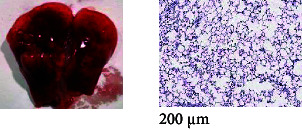	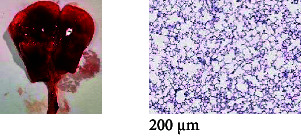

Kidney	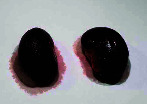	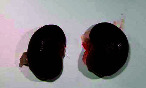

Spleen	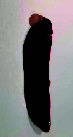	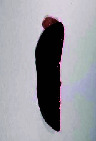

## Data Availability

The data that support the findings of this study are available from the corresponding author upon reasonable request.

## References

[1] Rungruangsaphakun J., Nakphaichit M., Keawsompong S. (2022). Nutritional Improvement of Copra Meal for Swine Feed. *Biocatalysis and Agricultural Biotechnology*.

[2] Pangsri P., Piwpankaew Y., Ingkakul A., Nitisinprasert S., Keawsompong S. (2015). Characterization of Mannanase From Bacillus Circulans NT 6.7 and its Application in Mannooligosaccharides Preparation as Prebiotic. *SpringerPlus*.

[3] Titapoka S., Keawsompong S., Haltrich D., Nitisinprasert S. (2008). Selection and Characterization of Mannanase-Producing Bacteria Useful for the Formation of Prebiotic Manno-Oligosaccharides From Copra Meal. *World Journal of Microbiology and Biotechnology*.

[4] Rungruangsaphakun J., Keawsompong S. (2018). Optimization of Hydrolysis Conditions for the Mannooligosaccharides Copra Meal Hydrolysate Production. *3 Biotech*.

[5] Sathitkowitchai W., Suratannon N., Keawsompong S. (2021). A Randomized Trial to Evaluate the Impact of Copra Meal Hydrolysate on Gastrointestinal Symptoms and Gut Microbiome. *PeerJ*.

[6] Kumar Suryawanshi R., Kango N. (2021). Production of Mannooligosaccharides from Various Mannans and Evaluation of Their Prebiotic Potential. *Food Chemistry*.

[7] Kovacs-Nolan J., Kanatani H., Nakamura A., Ibuki M., Mine Y. (2013). β-1,4-Mannobiose Stimulates Innate Immune Responses and Induces TLR4-Dependent Activation of Mouse Macrophages but Reduces Severity of Inflammation During Endotoxemia in Mice. *The Journal of Nutrition*.

[8] Ghosh A., Verma A. K., Tingirikari J. R., Shukla R., Goyal A. (2015). Recovery and Purification of Oligosaccharides From Copra Meal by Recombinant Endo-β-Mannanase and Deciphering Molecular Mechanism Involved and its Role as Potent Therapeutic Agent. *Molecular Biotechnology*.

[9] Salminen S., Collado M. C., Endo A. (2021). The International Scientific Association of Probiotics and Prebiotics (ISAPP) Consensus Statement on the Definition and Scope of Postbiotics. *Nature Reviews Gastroenterology and Hepatology*.

[10] Prayoonthien P., Nitisinprasert S., Keawsompong S. (2018). In Vitro Fermentation of Copra Meal Hydrolysate by Chicken Microbiota. *3 Biotech*.

[11] Sanz M. L., Côté G. L., Gibson G. R., Rastall R. A. (2006). Influence of Glycosidic Linkages and Molecular Weight on the Fermentation of Maltose-Based Oligosaccharides by Human Gut Bacteria. *Journal of Agricultural and Food Chemistry*.

[12] Gibson G. R., Hutkins R., Sanders M. E. (2017). Expert Consensus Document: The International Scientific Association for Probiotics and Prebiotics (ISAPP) Consensus Statement on the Definition and Scope of Prebiotics. *Nature Reviews Gastroenterology and Hepatology*.

[13] (2018). No, O. T. 423: Acute Oral Toxicity-Acute Toxic Class Method. *OECD Guidelines for the Testing of Chemicals, Section 2002, 4. Co-operation, O. F. E.; Development. Test No. 408: Repeated Dose 90-day Oral Toxicity Study in Rodents*.

[14] Council N. R. (2010). Recognition and Alleviation of Pain in Laboratory Animals.

[15] Thongsook T., Chaijamrus S. (2018). Optimization of Enzymatic Hydrolysis of Copra Meal: Compositions and Properties of the Hydrolysate. *Journal of Food Science and Technology*.

[16] Wang M., Niu J., Ou L., Deng B., Wang Y., Li S. (2019). Zerumbone Protects Against Carbon Tetrachloride (CCl4)-Induced Acute Liver Injury in Mice via Inhibiting Oxidative Stress and the Inflammatory Response: Involving the TLR4/NF-Κb/cox-2 Pathway. *Molecules*.

[17] Sundu B., Dingle J. (2003). Use of Enzymes to Improve the Nutritional Value of Palm Kernel Meal and Copra Meal.

[18] Qin Y., Wu X., Huang W. (2009). Acute Toxicity and Sub-Chronic Toxicity of Steroidal Saponins From *Dioscorea Zingiberensis* CH Wright in Rodents. *Journal of Ethnopharmacology*.

[19] Li Y., Kandhare A. D., Mukherjee A. A., Bodhankar S. L. (2019). Acute and Sub-Chronic Oral Toxicity Studies of Hesperidin Isolated From Orange Peel Extract in Sprague Dawley Rats. *Regulatory Toxicology and Pharmacology*.

[20] Jana U. K., Kango N. (2020). Characteristics and Bioactive Properties of Mannooligosaccharides Derived From Agro-Waste Mannans. *International Journal of Biological Macromolecules*.

[21] Kang S., Johnston T. V., Ku S., Ji G. E. (2020). Acute and Sub-Chronic (28-day) Oral Toxicity Profiles of Newly Synthesized Prebiotic Butyl-Fructooligosaccharide in ICR Mouse and Wistar Rat Models. *Toxicology Research*.

[22] Han Z.-Z., Xu H.-D., Kim K.-H. (2010). Reference Data of the Main Physiological Parameters in Control Sprague-Dawley Rats From Pre-Clinical Toxicity Studies. *Laboratory Animal Research: Home*.

[23] Olson H., Betton G., Robinson D. (2000). Concordance of the Toxicity of Pharmaceuticals in Humans and in Animals. *Regulatory Toxicology and Pharmacology*.

[24] Son C.-G., Han S.-H., Cho J.-H. (2003). Induction of Hemopoiesis by Saenghyuldan, a Mixture of Ginseng Radix, Paeoniae Radix Alba, and Hominis Placenta Extracts. *Acta Pharmacologica Sinica*.

[25] Adebayo J. O., Adesokan A. A., Olatunji L. A., Buoro D. O., Soladoye A. O. (2005). Effect of Ethanolic Extract of Bougainvillea Spectabilis Leaves on Haematological and Serum Lipid Variables in Rats.

[26] Kim S.-H., Ryu D.-S., Lee H.-S., Shin H.-R., Kwon J.-H., Lee D.-S. (2014). Acute Oral Toxicity of the Ethyl Acetate Fraction of Orostachys Japonicus in Mice. *Pharmaceutical Biology*.

[27] Wallach J. B. (2007). *Interpretation of Diagnostic Tests*.

[28] Féres C. a. o., Madalosso R., Rocha O. (2006). Acute and Chronic Toxicological Studies of Dimorphandra Mollis in Experimental Animals. *Journal of Ethnopharmacology*.

[29] Abdelghffar E. A., El-Nashar H. A. S., Al-Mohammadi A. G. A. (2021). Orange Fruit (Citrus Sinensis) Peel Extract Attenuates Chemotherapy-Induced Toxicity in Male Rats. *Food & Function*.

[30] Tennekoon K. H., Jeevathayaparan S., Kurukulasooriya A. P., Karunanayake E. H., Kurukulasooriya A. P., Karunanayake E. H. (1991). Effect of L-Carnitine Supplementation on Lead Acetate-Induced Liver Cell Apoptosis and Inflammation: Role of Caspase-3 and Glycogen Synthase Kinase-3β Enzymes. *Journal of Ethnopharmacology*.

[31] Seriana I., Akmal M., Darusman D., Wahyuni S., Khairan K., Sugito S. (2021). Neem Leaf (Azadirachta indica A. Juss) Ethanolic Extract on the Liver and Kidney Function of Rats. *The Scientific World Journal*.

[32] Li P., Wang S., Guan X. (2013). Acute and 13 Weeks Subchronic Toxicological Evaluation of Naringin in Sprague-Dawley Rats. *Food and Chemical Toxicology*.

[33] Jain M., Gote M., Dubey A. K. (2018). Safety Evaluation of Fructooligosaccharide (FOSSENCETM): Acute, 14-Day, and Subchronic Oral Toxicity Study in Wistar Rats. *Toxicology Research and Application*.

[34] Álvarez-Cervantes P., Izquierdo-Vega J. A., Morán-León J. (2021). Subacute and Subchronic Toxicity of Microencapsulated Pomegranate Juice in Rats and Mice. *Toxicology Research*.

[35] Abdel-Emam R. A., Ali M. F. (2022). Effect of L-Carnitine Supplementation on Lead Acetate-Induced Liver Cell Apoptosis and Inflammation: Role of Caspase-3 and Glycogen Synthase Kinase-3β Enzymes. *Life Sciences*.

[36] Tennekoon K. H., Jeevathayaparan S., Kurukulasooriya A. P., Karunanayake E. H. (1991). Possible Hepatotoxicity of Nigella Sativa Seeds and Dregea Volubilis Leaves. *Journal of Ethnopharmacology*.

[37] Seriana I., Akmal M., Darusman D., Wahyuni S., Khairan K., Sugito S. (2021). Neem Leaf (Azadirachta indica A. Juss) Ethanolic Extract on the Liver and Kidney Function of Rats. *The Scientific World Journal*.

[38] Li P., Wang S., Guan X. (2013). Acute and 13 Weeks Subchronic Toxicological Evaluation of Naringin in Sprague-Dawley Rats. *Food and Chemical Toxicology*.

[39] Intaratrakul K., Nitisinprasert S., Nguyen T.-H., Haltrich D., Keawsompong S. (2022). Manno-Oligosaccharides From Copra Meal: Optimization of its Enzymatic Production and Evaluation its Potential as Prebiotic. *Bioactive Carbohydrates and Dietary Fibre*.

[40] Prayoonthien P., Rastall R. A., Kolida S., Nitisinprasert S., Keawsompong S. (2019). In Vitro Fermentation of Copra Meal Hydrolysate by Human Fecal Microbiota. *3 Biotech*.

[41] Gadaga L. L., Tagwireyi D. (2014). Critical Review of the Guidelines and Methods in Toxicological Research in Africa. *Toxicological Survey of African Medicinal Plants*.

[42] Ochoa R. (2013). Pathology Issues in the Design of Toxicology Studies. *Haschek and Rousseaux's Handbook of Toxicologic Pathology*.

[43] Amresh G., Singh P. N., Rao C. V. (2008). Toxicological Screening of Traditional Medicine Laghupatha (Cissampelos Pareira) in Experimental Animals. *Journal of Ethnopharmacology*.

[44] Jain M., Gote M., Dubey A. K. (2018). Safety Evaluation of Fructooligosaccharide (FOSSENCETM): Acute, 14-Day, and Subchronic Oral Toxicity Study in Wistar Rats. *Toxicology Research and Application*.

[45] Kang S., You H. J., Lee Y.-G. (2020). Production, Structural Characterization, and In Vitro Assessment of the Prebiotic Potential of Butyl-Fructooligosaccharides. *International Journal of Molecular Sciences*.

